# Exploring the molecular and biological mechanisms of host response in chickens infected with highly pathogenic avian influenza virus (H5N1): An integrative transcriptomic analysis

**DOI:** 10.1371/journal.pone.0332689

**Published:** 2025-10-03

**Authors:** Sare Golpasand, Shahrokh Ghovvati, Zahra Pezeshkian

**Affiliations:** 1 Department of Animal Sciences, Faculty of Agriculture, University of Guilan, Rasht, Guilan, Iran; 2 BioGenTAC Inc., Technology Incubator of Agricultural Biotechnology Research Institute of Iran-North Branch (ABRII), Rasht, Iran; National Bureau of Animal Genetic Resources, INDIA

## Abstract

The H5N1 strain of highly pathogenic avian influenza (HPAI) has raised worldwide alarm, posing a threat to the health of both animals and humans. The study aimed to identify key genes and biological pathways modulated in chickens infected with H5N1 through meta-analysis. Four microarray datasets from GEO were normalized using RMA, Quantile methods, and the SVA package in RStudio. Differentially expressed genes (DEGs) were screened using the edgR package. Gene enrichment and network analyses were performed, and key genes in the protein-protein interaction (PPI) network were identified using the CytoHubba and MCODE plugins in Cytoscape. Additionally, to investigate the regulatory networks involved, we employed the miRDB web tool to identify microRNAs associated with each hub gene. The resulting miRNA-mRNA interaction network was constructed using Cytoscape software, providing insights into the post-transcriptional regulation of gene expression. Furthermore, WGCNA revealed co-expression modules tied to gene expression profiles. Gene expression analysis identified 259 DEGs between normal and infected lung tissues, with 140 up-regulated and 119 down-regulated genes (adj P-Value<0.05 and |log2 fold change (FC)| > 1). Ten hub genes (*EPSTI1, IFIH1, IFIT5, IRF1, IRF7, MX1, OASL, PARP14, RSAD2, USP18*) were identified. Up-regulated DEGs activated the host’s innate immune response, highlighting the roles of cytokines and chemokines in inflammation. The study showed that increased expression of interleukins, interferons, and growth factors in the extracellular space is influential for activating innate immunity mechanisms. These findings enhance the understanding of the molecular mechanisms underlying H5N1 infection in chickens.

## Introduction

The influenza virus belongs to the Orthomyxoviridae family, comprising five genera based on nucleoprotein (NP) and matrix (M) proteins [1]. Influenza B virus usually causes localized outbreaks, whereas influenza A virus is the main pathogen responsible for major influenza epidemics and pandemics. Avian influenza viruses (AIVs) frequently spill over from waterfowl reservoirs to humans, exhibit limited human-to-human transmissibility. The emergence of H5 strains, has posed substantial global health concerns. From 2019 to 2022, a comprehensive international assessment revealed a rapid shift in the dominant circulating subtype of H5 avian influenza from H5N8 to H5N1 [2]. Given the growing pandemic threat, it is critical to explore both existing diagnostic strategies and emerging approaches for prevention and treatment. Advances in in biotechnology and bioinformatics have facilitated the generation of large-scale biological datasets aimed at exploring key aspects of host–pathogen interactions. These datasets are now widely available through public repositories (GEO, Array Express, etc.), enabling further integrative analyses [3,4]. DNA microarray technology has become a widely adopted tool in influenza research for detecting genomic alterations and elucidating host-pathogen interactions [5–9]. Meta-analysis of gene expression data enhances statistical power, increases generalizability, and enables the identification of robust DEGs across independent studies. When combined with cross-platform normalization, such integrative approaches also improve reproducibility and reduce the need for repetitive animal experiments [10,11]. In avian influenza studies, meta-analyses have revealed a higher prevalence of the H5N1 subtype compared to H7N9, emphasizing its broader epidemiological threat [12,13]. The main objective of this research was to advance comprehension of the molecular mechanisms governing the host’s response to HPAIV. This insight could have significant implications for infection research, including early detection and treatment strategies.

## Materials and methods

### Data collection and preprocessing

**Fig 1:**
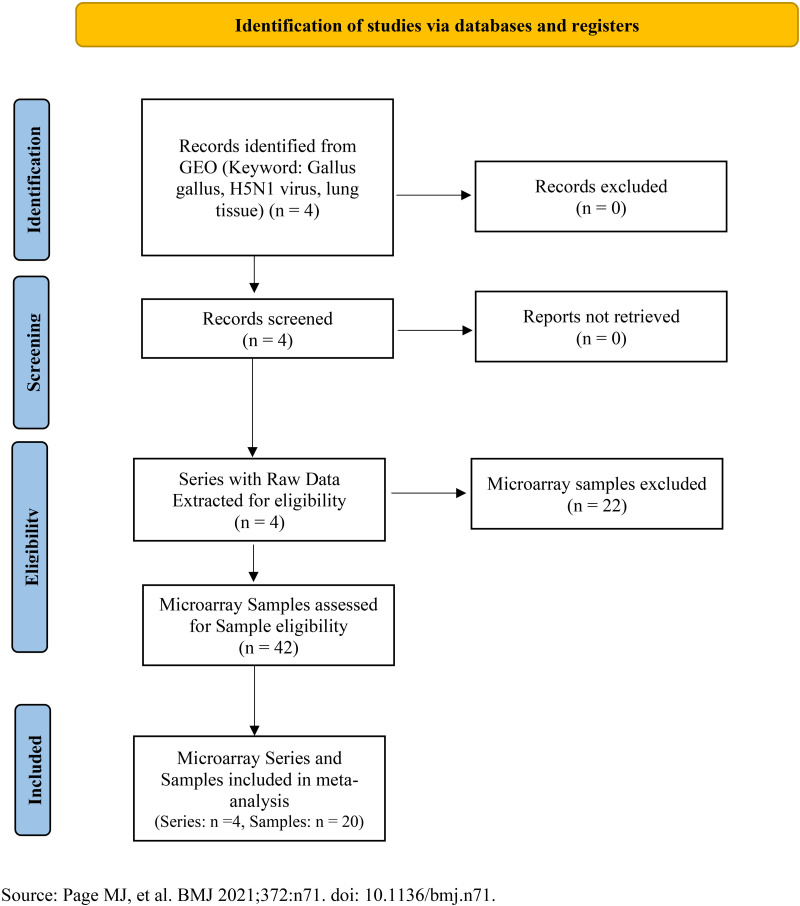
PRISMA flow diagram.

Several critical criteria were applied for dataset selection. These included sample size, number of replicates, and appropriate data distribution, among other relevant factors. Notably, among the investigated data, four microarray datasets comprising a total of 20 samples were identified, which were from different platforms and included normal lung tissue samples (7 control) and challenged with the H5N1 virus (13 challenged) in chickens, and were consistent with the search criteria of this research. Subsequently, these datasets were retrieved from the public database, the Gene Expression Omnibus (GEO) database of NCBI (http://www.ncbi.nlm.nih.gov/GEO) to facilitate further investigation and meta-analysis (S1 Table). The Affymetrix-based datasets GSE33389, GSE53930 and GSE53931 were normalized using the Robust Multi-Array Average (RMA) algorithm from the affy package, while the Agilent-based dataset GSE65231 was normalized using the Quantile method in RStudio software [14]. After pre-normalization of each dataset, gene expression values from duplicate rows across the datasets were averaged by aggregate function. The resulting data were then combined into a single matrix file using the merge function. To assess the quality of the integrated data and examine the distribution of samples, box plots and principal component analysis (PCA) were generated using the ggplot2 package of RStudio software [15]. PCA was performed to facilitate data visualization and outlier detection. A flow diagram following the PRISMA guidelines for systematic reviews and meta-analyses [16], illustrating the dataset selection process, is provided in Fig 1 and S1 Checklist.

### Data integration and batch effect removal

The integrated microarray data were normalized with the SVA package and the batch effects were removed using the ComBat function, an empirical Bayes cross-study normalization method implemented in the R software environment [17,18].

### Identification of differentially expressed genes

DEGs were identified by using the edgeR package of RStudio software. The criteria for identifying genes with differential expression were an absolute value of fold change greater than 1 and adj P-value less than 0.05. A volcano plot was also used to highlight gene expression differences between two comparison groups using SRplot [19].

### Gene ontology and KEGG pathway analysis

To explore the possible functions of the resulting DEGs, we implemented Gene Ontology (GO) and Kyoto Encyclopedia of Genes and Genomic (KEGG) pathway analysis using the Database for Annotation, Visualization and Integrated Discovery (DAVID)(https://david.ncifcrf.gov/home.jsp). GO terms refer to the following terms: Biological processes (BP), cellular components (CC), and molecular functions (MF) [20].

### Weight gene correlation network analysis (WGCNA)

WGCNA was employed to explore co-expression modules associated with the up or down-regulated gene expression profiles. The construction of gene co-expression networks, facilitated by the WGCNA R package in the iDEP tool, involved specific parameter settings. A soft threshold of 5 was applied, along with a minimum module size of 20, assigning unique color labels to each module [21].

### PPI network and module analysis

A PPI network was constructed from DEGs utilizing the Search Tool for the Retrieval of Interacting Genes/Proteins (STRING) database (https://string-db.org/), a comprehensive resource of experimentally validated and predicted PPI. This PPI network was then imported into Cytoscape software (Version 3.9.1) for further analysis to annotate functional interactions between DEGs and other associated genes [22]. To identify key functional modules within the network, the Molecular Complex Detection (MCODE) plugin was employed. MCODE facilitated the detection of significant modules by applying advanced parameters, including a degree cutoff of 2, a node score cutoff of 0.2, a k-core value of 2, and a maximum depth of 100.

### ClueGO/CluePedia functional analysis

To visualize GO categories and enrichment results of major modules identified by MCODE, the ClueGO and CluePedia Cytoscape plugin was used [23,24]. The ClueGO plugin could simplify the understanding of large and abundant gene sets by grouping important gene sets based on similarity.

### Identifying hub genes based on meta-analysis findings

The analysis of PPI networks was conducted using the CytoHubba plug-in based on the maximal clique centrality algorithm (MCC) within Cytoscape software to predict and investigate 10 key hub genes from all genes in the PPI network.

### Identification of the target miRNA and construction of the miRNA-mRNA regulatory network

We utilized the miRDB web tool to investigate and identify the target microRNAs (miRNAs) associated with each hub gene [25]. Additionally, the interaction network between miRNA and hub genes was constructed using Cytoscape software.

## Results

### Data collection and preprocessing

The integration of four microarray datasets, comprising a total of 20 samples, enabled a comprehensive meta-analysis that identified consistent gene expression patterns across different studies. Data preprocessing using RMA and Quantile algorithms improved the quality and reliability of the analysis, with values consistently falling within the positive range. The RMA algorithm was effective in background correction, quartile normalization, and data summarization to establish an appropriate scale for analysis [10].

### Data integration and batch effect removal

**Fig 2:**
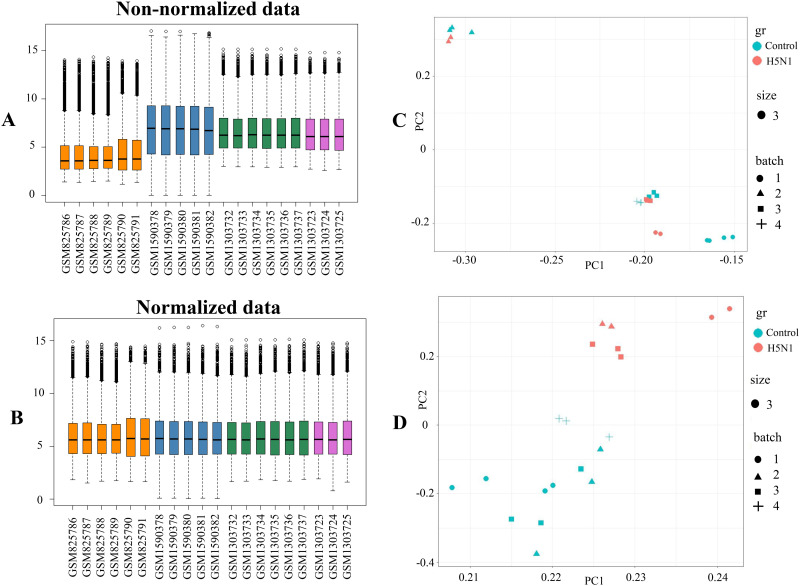
Box plots and PCA plots demonstrating non-normalized and normalized data of four microarray datasets. (A) Box plot of non-normalized data and (B) normalized data, illustrating the distribution of microarray datasets (GSE33389 in dark orange, GSE65231 in steel blue, GSE53931 in sea green, and GSE53930 in orchid). The central line within each box represents the median, and the box edges indicate the interquartile range (IQR). Vertical bracketed lines at both ends of the boxes signify the extreme values of the distribution. (C) PCA plot before normalization of datasets. The plot shows the dispersion of the samples and specifies how far and close the samples are from each other. The pink dots indicate the microarray data series of the control samples and the green dots indicate the data series challenged with H5N1. The shapes of circle, triangle, square and, plus sign indicate data sets number 1 to 4. (D) PCA plot after normalization of merged data. Pink dots (control samples) tend to be on the right side, and green dots (H5N1 challenged) tend to be on the left side. The X-axis represents the first dimension, and the Y-axis represents the second dimension. Marker shapes represent datasets 1 to 4 as in panel C.

Analysis revealed that 9911 genes were shared among the four datasets used for meta-analysis. Box plots and PCA were employed to assess the quality and sample grouping of the merged dataset (Fig 2A and C). The results revealed discrepancies in the median values depicted in the box plots of the microarray datasets. Additionally, the adequate segregation of samples from the control group and those subjected to HPAI was not accomplished in PCA. However, post-normalization PCA analysis demonstrated clear differentiation between HPAI-challenged and control samples. PCA models indicated appropriate quality of the merged microarray data for further scientific investigation (Fig 2B and D).

### Identification of differentially expressed genes

**Fig 3:**
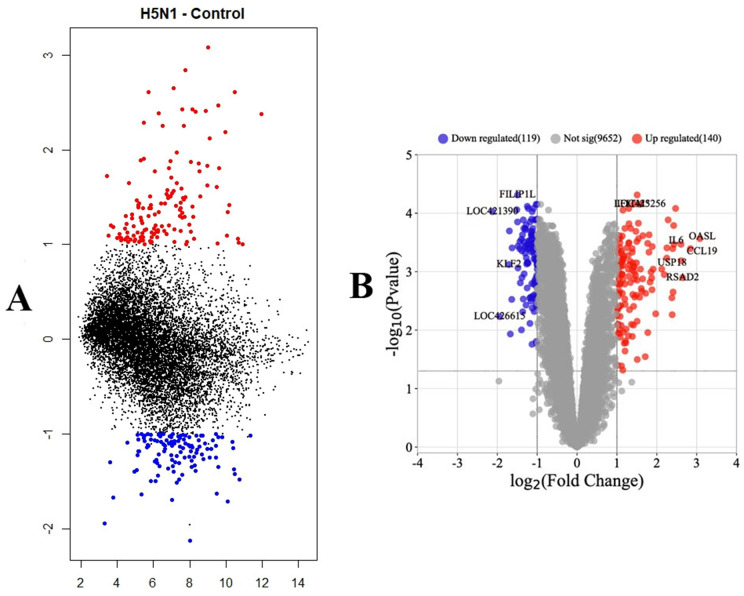
Mean difference and volcano plots of Differentially Expressed Genes (DEGs). (A) Mean Difference plot. The X-axis indicates the average logarithm of gene expression (mean), and the Y-axis indicates log2 fold change (FC) (differences). (B) Volcano plot of DEGs. Red dots show upregulated DEGs, blue dots show downregulated DEGs, and gray dots indicate gene expression with no significant difference in expression between the control and the challenged group.

The meta-analysis identified 259 DEGs between normal (control) and H5N1 HPAI-challenged tissues in chickens, with 140 genes up-regulated and 119 genes down-regulated (adj P-Value < 0.05 and |log2 fold change (FC)| > 1) (S2 Table). Visualization of the results and assessment of the magnitude of changes relative to the average, identified DEGs in lung tissue samples challenged with H5N1 HPAIV compared to control lung tissue. This analysis demonstrated that the number of genes with increased expression significantly exceeded the number of genes with decreased expression (Fig 3A). Also, the differential expression was visually represented, with volcano plots confirming a trend towards increased gene expression in challenged samples (Fig 3B).

### Gene ontology and KEGG pathway analysis

**Fig 4:**
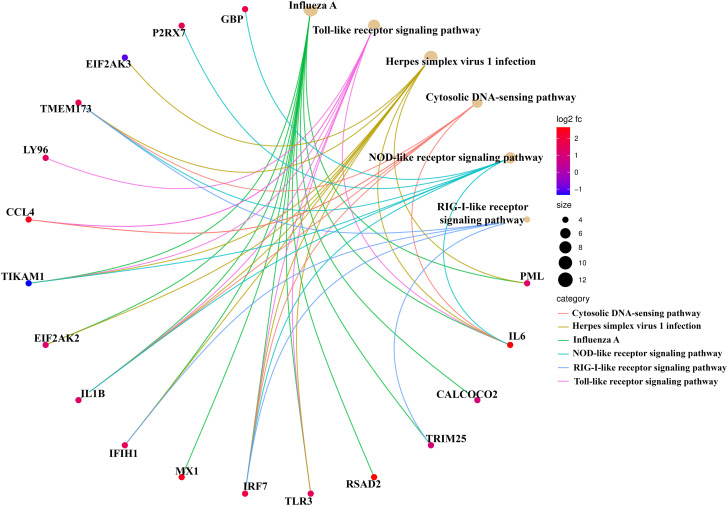
Enriched functional pathways of upregulated DEGs in chicken using SRplot, an online platform for data analysis and visualization.

Enrichment and ontology analysis of up-regulated genes indicated significant involvement in processes related to viral defense, including positive regulation of interferon production, inflammatory reactions, and Toll-like receptor (TLR) signaling (P < 0.05). Notable enrichment was also found in the extracellular space and transcription factor activity (Table 1). Down-regulated genes showed significant terms related to cellular responses to amino acid stimulus, protein localization, and GDP-dissociation inhibitor activity (P < 0.05) (Table 1). Enrichment analysis of DEGs revealed significant functions exclusively among upregulated DEGs, particularly in influenza type A-related pathways and host response to infection, involving 11 genes (Fig 4).

**Table 1:** Gene ontology pathways enriched by upregulated and downregulated DEGs in chicken.

Category	DEGs	Accession ID	Terms	Count	P-value	Genes
BP ^a^	Upregulated DEGs	GO:0051607	defense response to virus	7	3.68E-06	IFIH1, IL6, RSAD2, MX1, IRF7, TMEM173, TLR3
		GO:0032728	positive regulation of interferon-beta production	4	1.42E-04	IFIH1, IRF7, TMEM173, TLR3
		GO:0006954	inflammatory response	6	1.32E-03	PXK, IL1B, AGTR1, LOC417536, CCL19, TLR3
		GO:0009615	response to virus	4	1.37E-03	IFIH1, BATF3, IL1B, LOC417536
		GO:0032727	positive regulation of interferon-alpha production	3	3.40E-03	IFIH1, IRF7, TLR3
		GO:0071222	cellular response to lipopolysaccharide	4	3.97E-03	CD274, IL6, IL1B, CMPK2
		GO:0002224	toll-like receptor signaling pathway	3	1.49E-02	IL1B, IRF7, TLR3
		GO:0032760	positive regulation of tumor necrosis factor production	3	1.92E-02	IFIH1, IL6, TLR3
		GO:0032755	positive regulation of interleukin-6 production	3	2.58E-02	IFIH1, IL6, TLR3
		GO:0045087	innate immune response	4	3.46E-02	IFIH1, TRIM25, TMEM173, TLR3
		GO:0014850	response to muscle activity	2	3.54E-02	FNDC5, CAPN3
		GO:0002269	leukocyte activation involved in inflammatory response	2	3.54E-02	IL1B, CCL4
		GO:0006357	regulation of transcription from RNA polymerase II promoter	10	3.81E-02	MSX2, IRF1, TLX1, IRF7, FOXL2, PITX1, …
		GO:0051092	positive regulation of NF-kappaB transcription factor activity	3	4.31E-02	CAPN3, TRIM25, TLR3
		GO:0002232	leukocyte chemotaxis involved in inflammatory response	2	4.41E-02	IL1B, CCL4
		GO:0006955	immune response	4	4.53E-02	CD274, IL1B, CCL4, LOC417536
	Downregulated DEGs	GO:0071230	cellular response to amino acid stimulus	3	1.08E-02	RRAGC, CAPN2, SH3 BP4
		GO:1904778	positive regulation of protein localization to cell cortex	2	2.97E-02	GPSM2, GNAI1
		GO:0032392	DNA geometric change	2	2.97E-02	HMGB2, HMGB3
		GO:0051310	metaphase plate congression	2	3.94E-02	INCENP, GEM
CC ^b^	Upregulated DEGs	GO:0005615	extracellular space	15	5.62E-04	IL6, IFNB, IL8, SST, SFRP5, IL1B, CCL4, FGF18, COL9A3, CCL19, …
	Downregulated DEGs	GO:0031080	nuclear pore outer ring	2	2.86E-02	NUP85, NUP43
		GO:0005813	centrosome	5	3.95E-02	GPSM2, CCNJ, SASS6, MKS1, GNAI1
		GO:0005737	cytoplasm	23	4.30E-02	CCNJ, USP4, EIF4A3, HMGB2, RECQL, HMGB3, AMDHD1, HABP4, MKS1, …
MF ^c^	Upregulated DEGs	GO:0003700	transcription factor activity, sequence-specific DNA binding	6	1.01E-02	CEBPA, IRF1, IRF7, FOXL2, HNF4BETA, PITX1
		GO:0008009	chemokine activity	3	1.75E-02	CCL4, LOC417536, CCL19
		GO:0000981	RNA polymerase II transcription factor activity, sequence-specific DNA binding	9	3.41E-02	MSX2, IRF1, TLX1, IRF7, FOXL2, PITX1, …
	Downregulated DEGs	GO:0005092	GDP-dissociation inhibitor activity	2	2.67E-02	GPSM2, SH3 BP4
		GO:0042802	identical protein binding	8	3.27E-02	GPSM2, EIF2AK3, TRIP13, …
		GO:0016887	ATPase activity	5	3.96E-02	EIF4A3, RECQL, ATP1A1, TRIP13, SMC3

^a^Biological Processes; ^b^Cellular Components; ^c^Molecular Functions.

### Weight gene correlation network analysis

**Fig 5:**
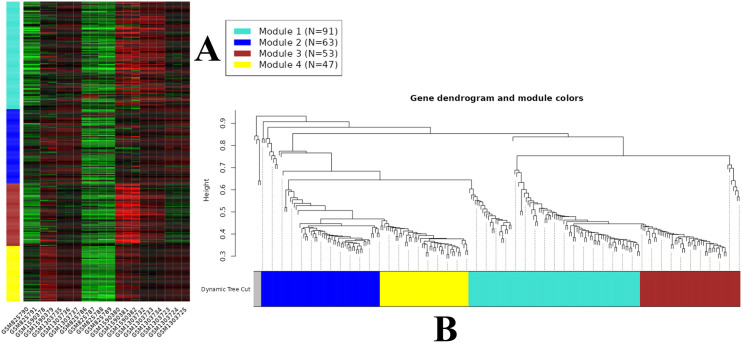
WGCNA-based gene co-expression network analysis. (A) Module partition trees of datasets. Modules are distinguished by different colors. The heatmap of modules related to each sample. In the heatmap of modules, left-margin annotation corresponds to module colors and their respective gene counts. Module 1 (turquoise) contains 91 genes, Module 2 (blue) contains 63 genes, Module 3 (brown) contains 53 genes, and Module 4 (yellow) consists of 47 genes. Gene upregulation is indicated in red, while downregulation is represented in green. This precise integration of information unveils distinct regulatory patterns of gene expression and diverse responses to the H5N1 virus challenge. (B) Gene dendrogram and module colors, derived from WGCNA output, present a hierarchical clustering of genes into four distinct modules, each characterized by unique cluster colors. This dendrogram provides a visual representation of the relationships and similarities among genes, while the assigned colors to the modules facilitate the identification and categorization of gene clusters.

The WGCNA algorithm is a highly utilized method for constructing gene co-expression networks and analyzing gene expression profiles. After hierarchical clustering and removal of five outlier genes, 254 genes were divided into four modules: blue, yellow, turquoise, and brown (Fig 5B). The distribution of genes among these modules is summarized in S3 Table. In addition, module gene expression was visualized as a heatmap in Fig 5A.

In our investigation, the turquoise module emerged as a focal point of interest, showcasing up-regulated genes such as *CD274*, *DBC1*, and *IL1B*. Concurrently, the blue module featured notable down-regulation in genes like *SLC7A6OS*, *SLU7*, and *GPSM2*. Within the brown module, a distinctive set of up-regulated genes, including *TMEM173*, *GIP*, and *PACSIN1*, underscored a coordinated immune response to the H5N1 AIV challenge. Conversely, the yellow module highlighted down-regulated genes like *ARMC6*, *TNFAIP8L1*, and *SGPL1*. By dissecting the co-expression networks, we can better identify how genes express in concert to mount an effective immune response, paving the way for more targeted and effective treatments.

### PPI network and module analysis

**Fig 6:**
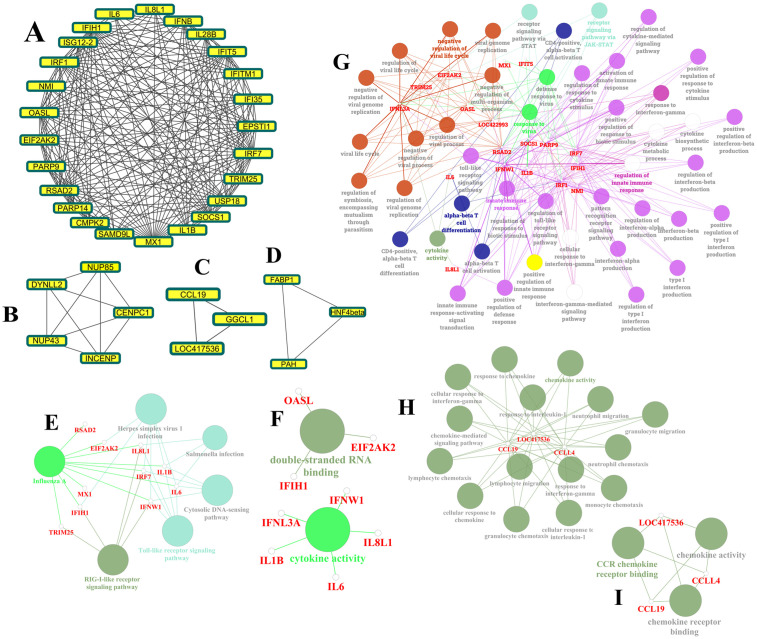
Identification of key modules of host response to HPAIV in chicken and their related enrichment paths and ontology. A: Module 1, B: Module 2, C: Module 3 and D: Module 4. E: functional enrichment pathways related to module 1, F: pathways related to molecular function in module 1, G: pathways activated in the biological processes section related to module 1, H: biological process pathways in module 3, and I: related pathways with molecular function in module 3. ClueGO/CluePedia extension of Cytoscape software was used to study functional enrichment and ontology of modules (P < 0.05). The bold fonts indicate the most important path of each group and the names of the related DEGs in each group are also displayed in red font.

To elucidate the coordinated response of genes to HPAIV infection, a PPI network was constructed using DEGs. MCODE plugin of Cytoscape identified four gene clusters (Fig 6A–D), with scores of 17.33, 5, 3, and 3, respectively. Most genes in the highest-scoring module were upregulated and associated with immune system activation and host defense responses, suggesting a prominent role in the antiviral response*.*

### ClueGO/CluePedia functional analysis

The results of ontology analysis and functional enrichment of Module 1, facilitated by the ClueGO/CluePedia plugin of Cytoscape, revealed that significant pathways were exclusively identified in Module 1 subsequent to gene enrichment. The functional enrichment analysis indicates that influenza type A, TLR messenger, and RIG-I receptor messenger pathways stand out as some of the most crucial functionally significant terms within Module 1. The upregulated genes within this module, including *MX1, IFIH1, RSAD2, IL1B*, and others, actively participate in these pathways (Fig 6E). GO analysis further emphasized cytokine activity and double-stranded RNA binding as critical molecular functions, crucial for viral detection and immune response (Fig 6F). In addition, network analysis identified significant processes related to the negative regulation of the viral life cycle and immune response regulation. These processes are integral in controlling viral replication and ensuring an effective immune response. The identification of alpha-beta T cell differentiation further emphasizes the importance of adaptive immunity, as these cells are essential for mounting a targeted and effective immune defense (Fig 6G). Chemokine activity plays a critical role in immune cell trafficking and in coordinating the immune response during infection (Fig 6H). The upregulation of genes within these modules highlights their biological significance, implicating them in both the physiological regulation of immune functions and the pathogenesis of diseases. Analysis of cellular components further identified chemokine receptor expression as a key feature (Fig 6I). Chemokine receptors (CCR) are expressed on essential cells involved in allergic inflammation, including eosinophils, basophils, lymphocytes, macrophages, and dendritic cells [26]. Overall, this analysis underscores the coordinated regulation of immune responses, providing insights into the mechanisms of disease resistance and susceptibility.

### Identifying hub genes based on meta-analysis findings

In this study, we conducted an exploration of important nodes within an interactome network utilizing the CytoHubba plugin, which integrates topological analysis algorithms. Of the eleven topological analysis methods implemented in CytoHubba, MCC demonstrated the highest accuracy in identifying essential proteins [27] and therefore, we utilized it for identifying essential nodes. The application of the CytoHubba yielded insightful results. The identified hub genes, including *EPSTI1, IFIH1, IFIT5, IRF1, IRF7, MX1, OASL, PARP14, RSAD2,* and *USP18*, emerged as central nodes in the PPI network (S1 Fig). These genes are significantly associated and are essential in activating the host’s immune system to mount an effective response against HPAI.

### Identification of target miRNA and construction of miRNA-mRNA regulatory network

**Fig 7:**
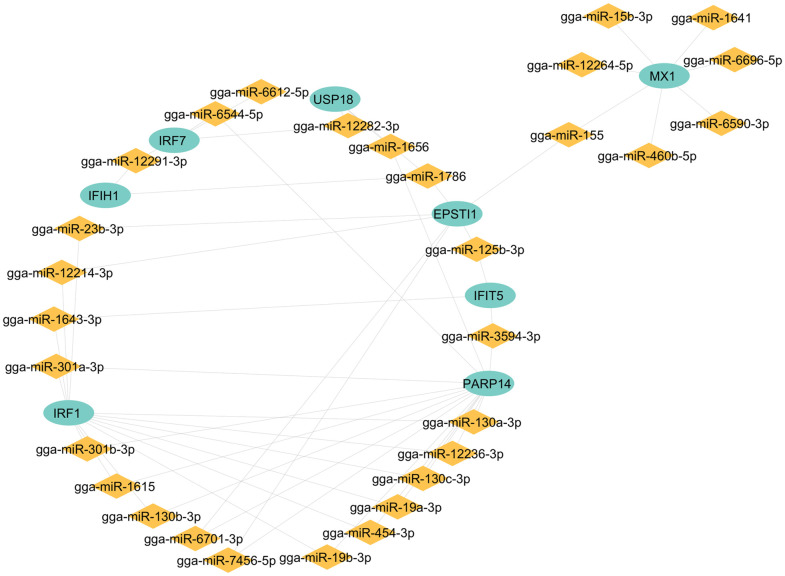
Interaction Between Hub Genes and Identified miRNAs. Depiction of miRNAs using yellow diamond shapes, while Hub genes are represented by green circles, respectively.

A comprehensive analysis was performed to identify miRNAs targeting hub genes using the miRdb database. To focus our investigation, we prioritized miRNAs with a well-documented role in infection and inflammation. To facilitate the visualization and interpretation of the complex miRNA-hub gene interaction network, we focused on the most frequently occurring interactions identified in our network analysis, which are visualized in Fig 7 and were generated using Cytoscape.

A complete list of identified 59 miRNAs and their interactions with hub genes can be found in S4 Table. Notably, several miRNAs, such as gga-miR-12243-3p and gga-miR-6701-3p, both targeting *EPSTI1*, have been previously implicated in immune responses to viral infections. *EPSTI1*, an interferon-stimulated gene, is significantly upregulated in macrophages upon IFNγ and lipopolysaccharide stimulation (LPS). Its silencing has been shown to enhance viral replication, underscoring its role in host defense mechanisms [28]. These miRNAs also participate in glutathione metabolism and immune modulation, impacting nutrient metabolism and signal transduction [29].

## Discussion

The HPAIV (H5N1), due to its high pathogenicity, zoonotic potential, and substantial economic losses, has received considerable global scientific attention. Consequently, research aimed at identifying genes involved in the host immune response remains essential for developing disease control strategies and advancing genetic selection for resistance. By leveraging advances in microarray technology, we conducted a meta-analysis of four microarray datasets from the GEO database, identifying 259 DEGs that enhance our understanding of the chicken immune response to H5N1. A key aspect of our study was the removal of batch effects using the SVA package, which employs surrogate variables and ComBat to adjust for hidden confounders and reduce technical variation [30–32]. This correction step is widely recognized for improving the robustness and reproducibility of gene expression analyses [33,34].

**Fig 8:**
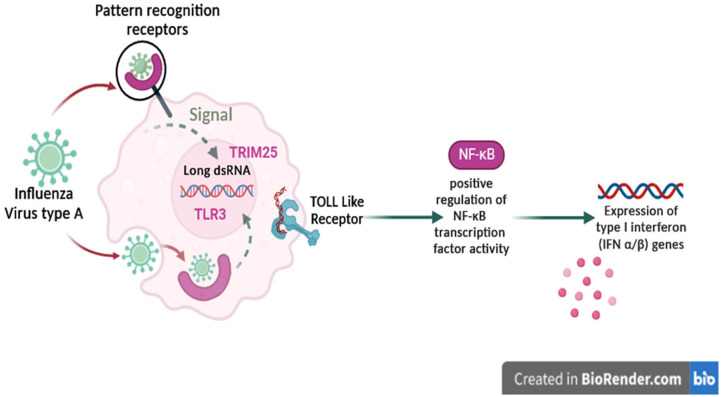
The response mechanism of the chicken innate immune system to type A influenza virus. Upon the entry of the influenza virus into the cell through endocytosis, it utilizes the host cell translation machinery for replication and packaging. Detection of surface antigens triggers signaling to the host cell nucleus, activating the immune response by enhancing gene expression and increasing cytokine production.

Enrichment and ontology analysis of the up-regulated genes indicated a strong association with biological processes related to defense response to viruses, regulation of alpha and beta interferon)*IFN-α* and *IFN-β*(production, inflammatory responses, and TLR signaling pathways. The immune response to H5N1 in chickens involves both innate and adaptive immunity. The innate immune system as the first line of defense, is activated upon recognition of pathogen-associated molecular patterns (PAMPs) by pattern recognition receptors (PRRs), such as TLRs. This recognition triggers both the transcription of antiviral genes and the release of type I interferons and pro-inflammatory cytokines (Fig 8). These findings confirm the established roles of these pathways in the innate immune response against viral infections [35,36].

Among the induced cytokines, **interleukin-1*β *(IL-1*β)* and *interleukin-6 (IL-6)* play pivotal roles in mediating inflammation and coordinating antiviral responses [37,38]. However, excessive cytokine and chemokine production, may trigger a cytokine storm, leading to tissue damage and impaired respiratory function. Such cytokine storms have been documented in both human and avian influenza infections, underscoring the need for targeted immunomodulatory therapies [39].

Type I interferons (*IFN-α* and *IFN-β*) act as key regulators of the innate antiviral response in chicken by regulating downstream immune pathways and restricting viral replication [40]. *IFN-α* is more effective than *IFN-β* in inducing interferon-stimulated genes (ISGs) and mounting robust antiviral defenses against vesicular stomatitis virus (VSV), Newcastle disease virus, and AIV [41]. Experimental studies using CRISPR/Cas9-mediated knockout of type I interferon receptor (*IFNAR1*) in chicken cells have further confirmed the critical role of *IFNAR1* signaling in combating H5N1 infection [42]. Moreover, the inflammatory response is further modulated by LPS, which activates leukocyte TLRs and stimulates the release of cytokines (*IL-1β*, *IL-6*, *TNF-α*) and chemokines (e.g., *IL-8*), thereby promoting vascular permeability and phagocyte migration in the avian immune system [43,44].

Expanding on these established mechanisms, our meta-analysis revealed significant upregulation of genes encoding cytokines, interleukins, and interferons in chickens infected with H5N1. These DEGs, predominantly active in the extracellular space, illustrate the host’s coordinated activation of both innate and adaptive immunity. Crucially, transcriptomic evidence demonstrated robust upregulation of JAK-STAT and NF-κB cascades downstream of type I interferons and TLRs, reinforcing their role as central antiviral sentinels.

Moreover, this study indicates that down-regulated DEGs were significantly enriched in amino acid metabolism pathways, reflecting a host-driven metabolic shift during infection. The upregulation of cytokines redirects amino acid resources toward immune cell proliferation and inflammatory protein synthesis, resulting in the suppression of growth-related processes [45]. Such reallocation may downregulate genes like *CAPN2*, which is involved in muscle development and has been previously linked to growth performance, thereby impairing growth in H5N1-infected chickens [46]. Similar metabolic adaptations have been linked to altered plasma amino acid levels, with methionine, glutamine, cysteine, and arginine playing roles in immune function across species [47,48].

The functional enrichment analysis revealed significant modulation of pathways, especially, influenza type A-related pathways. Influenza A virus relies on host proteases, particularly *TMPRSS2*, for activating hemagglutinin (HA), a critical step in viral entry. The essential role of *TMPRSS2* in promoting replication is underscored by evidence showing that its deletion confers resistance to influenza in mice [49]. Once internalized via endocytosis, the virus triggers immune pathways that can exacerbate pathology (S2 Fig). Our enrichment results underscore the engagement of canonical antiviral pathways, including TLR, JAK-STAT, and RIG-I, which are consistent with prior observations of innate antiviral responses in chickens challenged with H5N1 [50,51]. Notably, upregulation of *TLR3*, a key endosomal receptor, further supports the centrality of PRR-mediated recognition in the chicken antiviral defense [52].

We further employed WGCNA to explore gene co-regulation patterns in response to HPAIV infection. WGCNA complements differential-expression profiling by organizing transcripts into modules of highly correlated genes that often share common regulatory control or biological function [53,54]. This systems-level approach, revealed discrete gene modules indicative of coordinated immune activation. For instance, *CD274* and *IL1B*, key mediators of inflammatory signaling, were co-expressed and upregulated, whereas *GPSM2*, associated with cellular structural organization, was downregulated. These patterns revealed systemic host transcriptome restructuring, particularly in antiviral defense and immune regulator clusters.

Biological networks, including gene expression, PPI, and signaling pathways, form the basis of systems biology, offering integrated insights into molecular regulation and potential therapeutic targets [55,56]. Through key node analysis within the PPI network, we identified pivotal hub genes (*EPSTI1, IFIH1, IFIT5, IRF1, IRF7, MX1, OASL, PARP14, RSAD2*, and *USP18*), with significant implications for the host’s immune response to viral infections. The *MX1* gene, known for its antiviral activity in chickens, disrupts type A influenza viruses by targeting their lipid regions [57].

Studies show *MX1* gene expression impedes viral mRNA transcription and translation, particularly after induction by *IFN I* and *IFN III* [9]. Upregulation of *MX1* in lung tissue during H5N1 HPAIV challenge and its regulation in the ileum and submucosal lymph nodes highlight its critical role in the antiviral response [58]. *IFIH1*, encoding pivotal proteins in innate immunity, acts as a cytoplasmic receptor recognizing viral RNA and adapting the immune response [59]. Binding of viral RNA to *IFIH1* triggers a cascade releasing pro-inflammatory cytokines, notably interferons, which exhibit potent antiviral activity [60]. *IFIH1*’s role in enhancing immune cell activity against viral infections positions it as a significant candidate for developing targeted treatments against HPAIV. *IRF7*, a constituent of type I interferons, modulates the immune response to AIV infection in chickens. Overexpression of *IRF7* has been observed in chickens challenged with avian influenza, indicating its capacity to regulate various cellular processes within the host’s innate immune response [61,62]. Network analysis revealed upregulation of *IRF7* in chickens challenged with H5N1 HPAIV, underscoring its involvement in pivotal pathways related to influenza outbreaks and the host’s immune response. Furthermore, PPI network analysis revealed upregulation of oligoadenylate synthetase-like (*OASL*) following infection with various viruses, initiating a defense mechanism against infections. Studies on chickens exposed to the Newcastle virus demonstrated that targeting *OASL* holds promise for preventing and treating Newcastle disease in poultry [63].

Functional enrichment of Module 1 highlighted key antiviral pathways, including RIG-I-like receptor, TLR, and JAK–STAT signaling, as well as cytokine–cytokine receptor interactions. These results support the coordinated activation of innate immune responses in chickens during H5N1 infection. The upregulation of genes such as *MX1*, *IFIT5, OASL,* and *IFNB* reinforces the role of RIG-I-driven pathways in host defense, which is consistent with prior studies demonstrating enhanced antiviral responses in chicken cells upon RIG-I transduction [64,65]. Enrichment of cytokine-related terms, including chemokine activity and dsRNA binding, points to integrated signaling that regulates leukocyte recruitment and viral clearance. Notably, chemokines like *CCL4* have been implicated in viral restriction via metabolic modulation, suggesting immune–metabolic crosstalk during infection [66]. It can be interpreted that the genes present in the main modules contribute to key immunological pathways and may serve as valuable molecular entry points for the development of targeted vaccines, therapeutics, and breeding strategies. However, their functional relevance remains to be confirmed through targeted experimental validation.

Beyond transcriptional regulation, recent advances have highlighted the pivotal regulatory roles of non-coding RNAs, particularly circular RNAs (circRNAs) and miRNAs, in modulating gene expression across diverse species [67]. This study identified several miRNAs interacting with hub immune genes, suggesting an additional layer of regulation in the host response to H5N1 infection. Gga-miR-301a-3p interacts with both *IRF1* and *PARP14*, potentially modulating key components of the interferon signaling cascade. *IRF1*, a highly conserved transcription factor central to the induction of antiviral genes, is regulated by miRNAs such as gga-let-7f-3p and gga-miR-301b-3p, which fine-tune immune gene expression and cytokine signaling [68,69]. The regulatory complexity of this axis is further underscored by prior findings where members of the let-7 family, miR-155, and miR-146a modulate immune response genes during Salmonella infection [70–72], suggesting potential cross-pathogen roles in avian immunity. Similarly, *PARP14*, which enhances cytokine production via *IL-4* signaling, was associated with gga-miR-144-5p, gga-miR-153-5p, and gga-miR-1460, pointing to a multifaceted control mechanism involving both inflammatory and antiviral responses [73]. Additional miRNAs identified in this study, including gga-miR-12243-3p and gga-miR-6701-3p, target the interferon-stimulated gene *EPSTI1*, which plays a critical role in viral restriction. These interactions suggest miRNA-mediated modulation of genes responsible for interferon signaling and oxidative stress regulation [28].

Among the functionally characterized miRNAs, gga-miR-130b-3p and gga-miR-34b stand out as potential regulators of avian viral immunity. The former is known to inhibit IBDV replication, suppress inflammatory signaling during *Mycoplasma gallisepticum* infection, and reduce tumorigenesis in MDV-infected cells by targeting key oncogenes [74–76]. Its upregulation in IBDV models further supports its broad-spectrum antiviral potential [77]. Similarly, gga-miR-34b is upregulated in tracheal tissue of AIV-infected chickens, where it may influence B-cell differentiation and local immune tone [78]. Taken together, these findings illustrate a complex regulatory landscape in which miRNAs fine-tune host defense pathways in response to viral challenge. The selection of miRNAs, such as gga-miR-12243-3p, gga-miR-1582, gga-miR-1550-3p, and others, was based on their established regulatory roles in the literature, highlighting their potential in influencing host-pathogen interactions. These miRNAs offer promising insights for the diagnosis, prognosis, and treatment of AIV.

Collectively, integrated network and pathway analyses emphasize the cooperative action of interferon signaling, innate immune receptors, chemokine cascades, and metabolic reprogramming in the chicken response to H5N1. The identified hub genes and gene modules constitute promising biomarkers and candidate targets for antiviral drug development and genomic selection programs to enhance disease resilience in poultry. Future experimental validation, particularly functional studies using gene knock-out or over-expression models, will be essential to confirm the mechanistic roles of these candidates in HPAIV pathogenesis and host defense.

## Conclusions

The present integrative transcriptomic meta-analysis provides a systems-level perspective on host immune responses to H5N1 infection in chickens by minimizing batch effects and enhancing analytical rigor across multiple datasets. The analysis identified key hub genes (*EPSTI1, IFIH1, IFIT5, IRF1, IRF7, MX1, OASL, PARP14, RSAD2,* and *USP18*) implicated in innate immune signaling and antiviral defense. Systems-level interrogation identified cytokine-chemokine pathways as the cornerstone of host defense, with marked upregulation of pro-inflammatory mediators in H5N1-infected specimens (adj P-Value<0.05 and |log2 FC| > 1). Parallel transcriptome deconvolution affirmed hyperactivation of JAK-STAT and NF-κB signaling nodes downstream of TLR/type I IFN axes, positioning these pathways as master regulators of antiviral transcriptional reprogramming. These results establish a valuable framework for translating molecular insights into practical diagnostic solutions, targeted vaccines, breeding strategies, and genetic improvement to enhance avian resilience to HPAIV. Nonetheless, functional validation of the hub genes is necessary to realize the full potential of these discoveries in designing effective HPAIV mitigation strategies in avian.

## Supporting information

S1 Fig Hub genes from the protein-protein interaction (PPI) network analysis, ranked by centrality and visualized based on their interaction strength. (TIFF)

S2 Fig Influenza A pathway enriched with DEGs from H5N1-infected chickens. The diagram illustrates the molecular mechanisms triggered during H5N1 infection, highlighting key components of viral entry, replication, host pattern recognition, and downstream immune signaling. The red to green chromatogram in the picture shows the amount of gene fold changes (logFC), the red color shows more logFC, and the genes marked with red color also have a key role in the type A influenza pathway.
(TIFF)

S1 Checklist PRISMA 2020 Checklist. (PDF)

S1 Table Microarray data information related to H5N1 HPAIV employed in the study. (DOCX)

S2 Table List of differentially expressed genes including 140 up-regulated genes and 119 down-regulated genes which were identified between healthy and H5N1 influenza virus infected lung tissue samples in chickens. (DOCX)

S3 Table List of the genes of each module. (DOCX)

S4 Table Hub genes and miRNA list. (DOCX)
