# Supplementary figures and images for: Exploring the molecular and biological mechanisms of host response in chickens infected with highly pathogenic avian influenza virus (H5N1): An integrative transcriptomic analysis
Source: PLoS One. 2025 Oct 3;20(10):e0332689. doi: 10.1371/journal.pone.0332689 (PMC12494259; doi:10.1371/journal.pone.0332689)

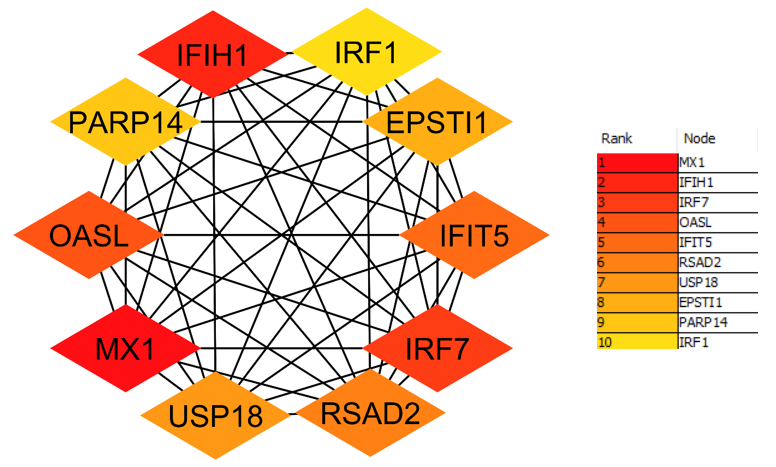

Supplement: S1 Fig — (TIFF) [file pone.0332689.s001.tiff]

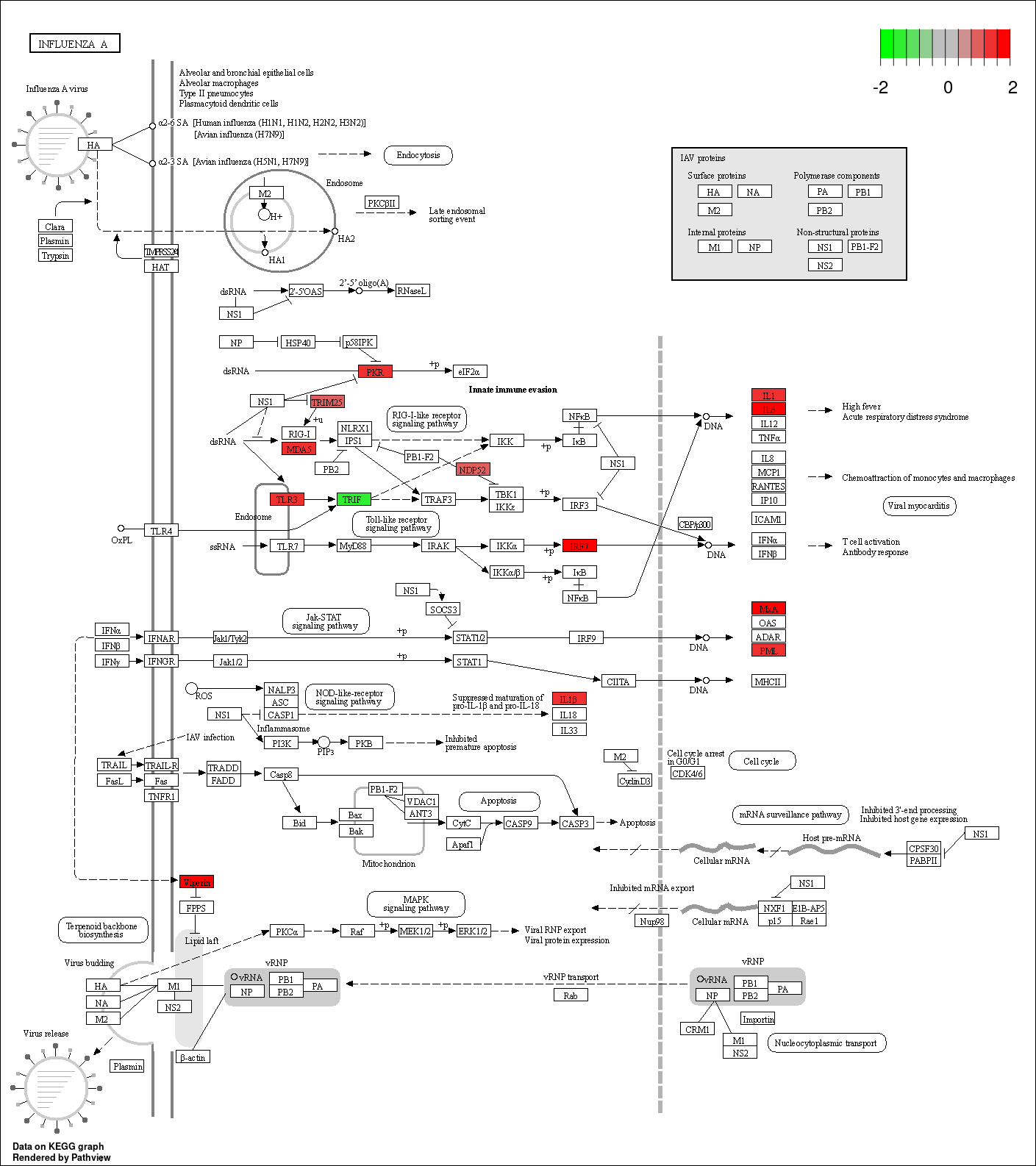

Supplement: S2 Fig — The diagram illustrates the molecular mechanisms triggered during H5N1 infection, highlighting key components of viral entry, replication, host pattern recognition, and downstream immune signaling. The red to green chromatogram in the picture shows the amount of gene fold changes (logFC), the red color shows more logFC, and the genes marked with red color also have a key role in the type A influenza pathway. (TIFF) [file pone.0332689.s002.tiff]
