# Supplementary material for: Exploring the molecular and biological mechanisms of host response in chickens infected with highly pathogenic avian influenza virus (H5N1): An integrative transcriptomic analysis
Source: PLoS One. 2025 Oct 3;20(10):e0332689. doi: 10.1371/journal.pone.0332689 (PMC12494259; doi:10.1371/journal.pone.0332689)
Supplement: S1 Table — (DOCX) [file pone.0332689.s004.docx]

| **S1 Table-** Microarray data information related to H5N1 HPAIV employed in the study. | | | |
| --- | --- | --- | --- |
| **Reference**  **Study** | **Accession Number** | **Platform** | **GEO accession** |
| [1-3] | [GSM825786](https://www.ncbi.nlm.nih.gov/geo/query/acc.cgi?acc=GSM825786)  [GSM825787](https://www.ncbi.nlm.nih.gov/geo/query/acc.cgi?acc=GSM825787)  [GSM825788](https://www.ncbi.nlm.nih.gov/geo/query/acc.cgi?acc=GSM825786)  [GSM825789](https://www.ncbi.nlm.nih.gov/geo/query/acc.cgi?acc=GSM825787)  [GSM825790](https://www.ncbi.nlm.nih.gov/geo/query/acc.cgi?acc=GSM825787)  [GSM825791](https://www.ncbi.nlm.nih.gov/geo/query/acc.cgi?acc=GSM825787) | Affymetrix Chicken Genome Array | GSE33389 |
| [4] | [GSM1590378](https://www.ncbi.nlm.nih.gov/geo/query/acc.cgi?acc=GSM1590378)  [GSM1590379](https://www.ncbi.nlm.nih.gov/geo/query/acc.cgi?acc=GSM1590378)  [GSM1590380](https://www.ncbi.nlm.nih.gov/geo/query/acc.cgi?acc=GSM1590378)  [GSM1590381](https://www.ncbi.nlm.nih.gov/geo/query/acc.cgi?acc=GSM1590378)  [GSM1590382](https://www.ncbi.nlm.nih.gov/geo/query/acc.cgi?acc=GSM1590378) | Agilent-059389 Custom Chicken Gene Expression 8X60k | GSE65231 |
| [5] | [GSM1303732](https://www.ncbi.nlm.nih.gov/geo/query/acc.cgi?acc=GSM1303732)  [GSM1303733](https://www.ncbi.nlm.nih.gov/geo/query/acc.cgi?acc=GSM1303732)  [GSM1303734](https://www.ncbi.nlm.nih.gov/geo/query/acc.cgi?acc=GSM1303732)  [GSM1303735](https://www.ncbi.nlm.nih.gov/geo/query/acc.cgi?acc=GSM1303732)  [GSM1303736](https://www.ncbi.nlm.nih.gov/geo/query/acc.cgi?acc=GSM1303732)  [GSM1303737](https://www.ncbi.nlm.nih.gov/geo/query/acc.cgi?acc=GSM1303732) | Affymetrix Chicken Genome Array | GSE53931 |
|  | [GSM1303723](https://www.ncbi.nlm.nih.gov/geo/query/acc.cgi?acc=GSM1303723)  [GSM1303724](https://www.ncbi.nlm.nih.gov/geo/query/acc.cgi?acc=GSM1303723)  [GSM1303725](https://www.ncbi.nlm.nih.gov/geo/query/acc.cgi?acc=GSM1303723) | Affymetrix Chicken Genome Array | GSE53930 |

1. Kuchipudi SV, Dunham SP, Chang K-C. DNA microarray global gene expression analysis of influenza virus-infected chicken and duck cells. Genomics data. 2015;4:60-4.

2. Kuchipudi SV, Tellabati M, Sebastian S, Londt BZ, Jansen C, Vervelde L, et al. Highly pathogenic avian influenza virus infection in chickens but not ducks is associated with elevated host immune and pro-inflammatory responses. Veterinary research. 2014;45(1):1-18.

3. Chothe SK, Nissly RH, Lim L, Bhushan G, Bird I, Radzio-Basu J, et al. NLRC5 serves as a pro-viral factor during influenza virus infection in chicken macrophages. Frontiers in cellular and infection microbiology. 2020;10:230.

4. Ranaware PB, Mishra A, Vijayakumar P, Gandhale PN, Kumar H, Kulkarni DD, et al. Genome wide host gene expression analysis in chicken lungs infected with avian influenza viruses. PLoS One. 2016;11(4):e0153671.

5. Hu J, Mo Y, Wang X, Gu M, Hu Z, Zhong L, et al. PA-X decreases the pathogenicity of highly pathogenic H5N1 influenza A virus in avian species by inhibiting virus replication and host response. Journal of virology. 2015;89(8):4126-42.
