# Supplementary material for: Exploring the molecular and biological mechanisms of host response in chickens infected with highly pathogenic avian influenza virus (H5N1): An integrative transcriptomic analysis
Source: PLoS One. 2025 Oct 3;20(10):e0332689. doi: 10.1371/journal.pone.0332689 (PMC12494259; doi:10.1371/journal.pone.0332689)
Supplement: S2 Table — (DOCX) [file pone.0332689.s005.docx]

| **S2 Table-** List of differentially expressed genes including 140 up-regulated genes and 119 down-regulated genes which were identified between healthy and H5N1 influenza virus infected lung tissue samples in chickens. | | |
| --- | --- | --- |
| **Gene Symbols** | | **DEGs** |
| LOC771089, LOC423256, IFITM5, LOC415756, PML, PSTPIP2, LOC418168, P2RX7, LOC771028, CCL4, ZP1, LOC420107, LOC424265, GBP, LINGO1, TMEM173, PXK, DBC1, B3GNT7, OASL, CEBPA, IRF10, LOC419812, CD274, CRISPLD2, SAMD9L, IL6, IFIT5, FAM26F, XDH, MAB21L2, LOC417192, CCL19, CMPK2, LY96, LOC427201, FOXL2, LYG2, MAFB, NCF2, COL9A3, TM4SF19, BATF3, LOC423478, CAPN3, PIGA, USP18, LOC418423, EAF2, IFI35, PARP9, OGFR, CALCOCO2, LOC771972, LOC415325, HNF4beta, BAIAP2L2, LOC416147, RLN3, TRIM25, KLHL10, GZMA, LOC396260, GADD45B, IRG1, FZD5, VCAM1, IL13RA2, SFRP5, TOX3, FAM149A, LOC420108, FGF18, GKAP1, RSAD2, EPSTI1, PITX1, AGTR1, OLFML1, BCMO1, TLR3, IRF7, IRF1, GBE, LOC422513, IL28B, PTPRN2, SNX10, LOC422993, LOC417536, EFHD1, MX1, OTP, CCKBR, IFNB, LOC431323, TGM4, MITD1, LHX3, NT5C3L, RASD1, ISG12-2, CHRNA6, LRRC39, CHODL, ATP8A1, ASB11, PACSIN1, EPB42, GIP, LIPA, GCH1, K123,ADC, SCG2, IFIH1, PAH, ZC3HAV1, GLRX, NMI, LOC693249, KCNJ5, P2RX3, MYL4, F3, TLX1, PARP14, IL1B, LOC420381, GPR20, EIF2AK2, FABP1, SST, FNDC5, IL8, SOCS1, HSP25, SERPINB10, LOC422654 | | **Upregulated DEGs** |
| FILIP1, EED, DCBLD1, HMGB3, LOC425015, DENND2A, TNFAIP8L1, MYH10, LOC421390, FNIP2, PRRG1, CCDC58, SPTBN1, BAMBI, GAS6, ING2, CDR2, RAMP3, TMEM120B, RRP7A, SPRY2, SH3BP4, TYRO3, CMAS, SETD6, RBM15B, NUP35, TSPAN6, RAB12, CAV2, TFB2M, RRAGC, GPSM2, PDDC1, NUP43, MTRF1L, AASDHPPT, USP12, TP53RK, TICAM1, R3HCC1, RMND5A, LOC421975, PRIM1, ARHGAP10, BRMS1L, ACAP2, EIF2AK3, ORAI1, YWHAH, XRCC2, GINS3, LRRCC1, DYNLL2, SIPA1L1, AMDHD1, TMEM26, TDP1, TMEM188, LOC417094, CENPC1, SASS6, PVRL3, WEE1, SLC25A12, CXCR7, HABP4, SGPL1, TRIP13, TRIM69, MKS1, TMEM171, TMEM55A, ATP1A1, FAM98A, TEX10, BBS12, ID4, KLF2, SNX30, OSBPL2, BET1, POP4, DCUN1D4, EIF4A3, WNT2B, NUP85, ARMC6, CNKSR3, CAB39, INCENP, TARDBP, NEK3, RSRC2, ZFYVE21, SLC7A6OS, USP4, POLR2B, SMC3, RFTN1, TMF1, SLU7, CAPN2, CCNJ, PNRC2, NSMCE4A, ROR1, GNAI1, NES, ISY1, RECQL, GEM, LOC426615, TMEM196, LOC421332, LOC431003, ADCY8, HMGB2, CA4 | | **Downregulated DEGs** |
|  |  |  |
