# Supplementary material for: Exploring the molecular and biological mechanisms of host response in chickens infected with highly pathogenic avian influenza virus (H5N1): An integrative transcriptomic analysis
Source: PLoS One. 2025 Oct 3;20(10):e0332689. doi: 10.1371/journal.pone.0332689 (PMC12494259; doi:10.1371/journal.pone.0332689)
Supplement: S3 Table — (DOCX) [file pone.0332689.s006.docx]

| **S3 Table-** List of the genes of each module. | | |
| --- | --- | --- |
| Module color | Module  number | Symbol of Genes |
| turquoise | 1 | CD274, DBC1, IL1B, MYL4, MKS1, GINS3, KCNJ5, PML, IRF1, RRAGC, CALCOCO2, LHX3, LOC415325, AZIN2, KLHL10, NT5C3L, TOX3, P2RX7, LOC417192, ORAI1, RSRC2, EPB42, VCAM1, BCMO1, FN3, COL9A3, CRISPLD2, LIPA, PXK, B3GNT7, TYRO3, SIPA1L1, EIF2AK2, HMGB2, SNX10, IFIH1, LOC423478, GBE, PARP14, PARP9, NMI, GKAP1, PAH, SERPINB10, USP18, FAM149A, ISG12-2, OASL, ZP1, ATP8A1, FILIP1L, CHODL, FABP1, MX1, GPR20, MITD1, LYG2, EPSTI1, NEK3, LOC420381, BATF3, CAPN3, EAF2, EFHD1, WNT2B, GGCL1, CHRNA6, HNF4beta, AGTR1, LOC422654, LOC417536, LOC769329, TICAM1, CMPK2, LY96, WEE1, CEBPA, CCL19, GLRX, POP4, K123, IL8, LOC396260, LOC417094, LOC424265, LOC771972, MAFB, OGFR, PRIM1, R3HCC1, TRIM69 |
| blue | 2 | SLC7A6OS, SLU7, GPSM2, FGF18, OLFM1, NUP35, LINGO1, USP4, KLF2, SH3BP4, TP53RK, SASS6, CA4, DYNLL2, PITX1, PTPRN2, CCNJ, ACAP2, SPTBN1, HMGB3, CAV2, FNIP2, ARHGAP10, IL6, ROR1, AMDHD1, ZFYVE21, CENPC1, DENND2A, MTRF1L, EED, DCUN1D4, LOC431003, LOC426615, DCBLD1, ATP1A1, MSX1, TMEM171, SNX30, PRRG1, LRRC1, GAS6, SPRY2, USP12, TMEM196, PDDC1, TMEM120B, PNRC2, SCG2, BET1, SETD6, CNKSR3, TMEM26, CDR2, RAB12, MAB21L2, TMEM55A, ID4, ISY1, LOC421390, LOC771028, RBM15B, TMEM188 |
| brown | 3 | TMEM173, GIP, PACSIN1, TRIM25, FNDC5, NCF2, LOC771089, RASD1, LRRC39, TM4SF19, IFIT5, FOXS1, SOCS1, SFRP5, TLX1, XDH, LOC423256, SAMD9L, TGM4, GCH1, BAIAP2L2, LOC418168, ZC3HAV1, ARHGEF28, FAM26F, RLN3, RSAD2, PIGA, IRG1, GZMA, IL13RA2, FOXL2, LOC420107, IRF7, PSTPIP2, GADD45, OTP, GBP, CCKBR, TLR3, FZD5, HSP25, IFI35, LOC415756, LOC416147, LOC418423, LOC419812, LOC420108, LOC422513, LOC422993, LOC431323, NES, P2RX3 |
| yellow | 4 | ARMC6, TNFAIP8L1, SGPL1, OSBPL2, YWHAH, TSPAN6, INCENP, NUP85, GNAI1, SMC3, CAPN2, NSMCE4A, SLC25A12, BRMS1L, FAM98A, TFB2M, ING2, TDP1, BBS12, RRP7A, NUP43, TRIP13, HABP4, RECQL, CMAS, TMF1, TEX10, CCDC58, MYH10, RMND5A, GEM, EIF2AK3, ADCY8, C2orf44, POLR2B, EIF4A3, LOC425015, XRCC2, RAMP3, BAMBI, AASDHPPT, CAB39, TARDBP, CXCR7, RFTN1, LOC421332, PVRL3 |
