# Supplementary material for: Exploring the molecular and biological mechanisms of host response in chickens infected with highly pathogenic avian influenza virus (H5N1): An integrative transcriptomic analysis
Source: PLoS One. 2025 Oct 3;20(10):e0332689. doi: 10.1371/journal.pone.0332689 (PMC12494259; doi:10.1371/journal.pone.0332689)
Supplement: S4 Table — (DOCX) [file pone.0332689.s007.docx]

| **S4 Table-** Hub genes and miRNA list. | |
| --- | --- |
| **Gene Symbol** | **miRNA name** |
| EPSTI1 | gga-miR-12243-3p, gga-miR-1582, gga-miR-12214-3p , gga-miR-1550-3p, gga-miR-6701-3p, gga-miR-7457-3p |
| IFIH1 | gga-miR-9b-3p, gga-miR-34b-5p, gga-miR-142-5p, gga-miR-26a-5p, gga-miR-26a-2-5p, gga-miR-12210-5p, gga-miR-1786, gga-miR-7442-5p |
| IFIT5 | gga-miR-7453-3p, gga-miR-7452-3p, gga-miR-460a-5p |
| IRF1 | gga-let-7f-3p, gga-let-7a-3p, gga-let-7k-3p, gga-miR-148a-3p, gga-miR-148b-3p , gga-miR-454-3p , gga-miR-12214-3p, gga-miR-130c-3p, gga-miR-301a-3p, gga-miR-130a-3p, gga-miR-301b-3p, gga-miR-130b-3p, gga-miR-23b-3p, gga-miR-301b-5p, gga-miR-466, gga-miR-1812-5p, gga-miR-6560-5p, gga-miR-1397-5p, gga-miR-1643-3p, gga-miR-219b |
| IRF7 | gga-miR-6661-5p, gga-miR-1791-5p, gga-miR-1723 |
| MX1 | gga-miR-155, gga-miR-460b-5p |
| PARP14 | gga-miR-144-5p, gga-miR-153-5p, gga-miR-1460, gga-miR-130c-3p, gga-miR-6701-3p, gga-miR-454-3p, gga-miR-301b-3p, gga-miR-130b-3p, gga-miR-130a-3p, gga-miR-301a-3p |
| USP18 | gga-miR-1806, gga-miR-7439-3p, gga-miR-12222-3p, gga-miR-6625-5p, gga-miR-6631-5p, gga-miR-1786, gga-miR-6589-5p |
